# Giving Good Directions: Order of Mention Reflects Visual Salience

**DOI:** 10.3389/fpsyg.2015.01793

**Published:** 2015-12-09

**Authors:** Alasdair D. F. Clarke, Micha Elsner, Hannah Rohde

**Affiliations:** ^1^School of Psychology, The College of Life Sciences and Medicine, University of Aberdeen Aberdeen, UK; ^2^Department of Linguistics, The Ohio State University Columbus, OH, USA; ^3^Linguistics and English Language, University of Edinburgh Edinburgh, UK

**Keywords:** referring expressions, visual search, visual salience

## Abstract

In complex stimuli, there are many different possible ways to refer to a specified target. Previous studies have shown that when people are faced with such a task, the content of their referring expression reflects visual properties such as size, salience, and clutter. Here, we extend these findings and present evidence that (i) the influence of visual perception on sentence construction goes beyond content selection and in part determines the order in which different objects are mentioned and (ii) order of mention influences comprehension. Study 1 (a corpus study of reference productions) shows that when a speaker uses a relational description to mention a salient object, that object is treated as being in the common ground and is more likely to be mentioned first. Study 2 (a visual search study) asks participants to listen to referring expressions and find the specified target; in keeping with the above result, we find that search for easy-to-find targets is faster when the target is mentioned first, while search for harder-to-find targets is facilitated by mentioning the target later, after a landmark in a relational description. Our findings show that seemingly low-level and disparate mental “modules” like perception and sentence planning interact at a high level and in task-dependent ways.

## 1. Introduction

When referring to an entity (the *target*) in a visual scene, speakers often describe it relative to some nearby *landmark*: “the woman next to the stairs.” Previous research demonstrates that speakers choose these landmarks with reference to the visual properties of the scene, and in particular that they prefer those that are larger and easier to see (Kelleher et al., 2005; Duckham et al., 2010; Clarke et al., 2013). Much less is known about how these perceptual effects extend to the information-structural ordering of elements in a description. Although alternative orders are available (“next to the stairs is a woman”), most existing models of reference do not address the production format question: how speakers choose to package the content of a referring expression when it includes both a target and one or more disambiguating landmarks. In this work, we demonstrate via a corpus study of reference productions that visual perception influences the order chosen: larger and more visually salient landmarks are more likely to precede the target. The results from a subsequent comprehension study using a visual search task show that this pattern of ordering also helps the listener to find the target faster. The production and comprehension results indicate that dialogue participants' perceptions of the scene have far-reaching effects on both referring expression generation (REG) and understanding. Visual perception is not confined to providing inputs to a content selection mechanism, as in many popular models, but also contributes toward high-level decisions about the expression's structure.

Theories which acknowledge a role for perception in ordering the description do so in two ways. In least-effort theories, speakers compose references using cognitively inexpensive heuristics (Beun and Cremers, 1998). In particular, speakers order large objects first because they see them earliest. Such an approach is in line with egocentric models of production in which speakers use what they are familiar with to estimate what objects may be visible and shared (Horton and Keysar, 1996). Neo-Gricean theories, on the other hand, treat ordering preferences as an example of *audience design*, in which speakers construct referring expressions which will help their listeners find the target quickly and easily. Thus, one critical prediction of the neo-Gricean approach is that such speaker behavior is actually helpful for listeners.

Our visual search study shows that this is in fact the case: listeners find the target object faster when a highly salient landmark is referred to earlier rather than later, and when a difficult-to-see landmark is referred to later rather than earlier. Thus, neo-Gricean theories remain a viable explanation for the ordering preference. In particular, the pattern fits neatly into more general theories of *information structure* which state that given (familiar) information typically precedes new information in the sentence (Prince, 1981; Ward and Birner, 2001). Although many researchers have stated that perceptually salient entities can be treated as familiar by discourse participants (Ariel, 1988; Roberts, 2003), few have given a detailed account of the kinds of perceptual factors which contribute. Cognitive semantics defines partitions in cognitive semantics between figure and ground (Talmy, 1978): Figures are elements that are smaller or less immediately perceivable (visual salience) and of greater concern or relevance (task salience), while Ground is likely to be larger, more immediately perceivable, and more familiar. Although the work on figure and ground indicates how elements in complex descriptions relate, it does not specify which orderings are preferred in production or comprehension. Here we show, in line with prior work on information structure and the on distinction between figure and ground, that computational models of visual salience correctly predict which objects speakers are likely to place earlier in their descriptions. Furthermore, listeners are found to be sensitive to order of mention, showing facilitation when a target that is easy to find is mentioned first and also when a hard-to-find target is preceded by a mention of a more salient easy-to-find landmark.

Earlier studies evaluating automatically generated referring expressions have shown that the most human-like ones are not always the most helpful for listeners (Belz and Gatt, 2008), suggesting that at least some tendencies in human REG do not involve clear estimates of listener needs. Our results imply that information structural patterns are not among them, and on the contrary may even be the product of deliberate optimization. Moreover, although systems for automatic REG have given little attention to ordering in the past, our results suggest that the use of perceptual data may lead to both more human-like references and better performance.

## 2. Motivation

Humans are highly proficient at REG, and human-like performance is often taken as a goal for automatic REG systems (Viethen and Dale, 2006). But more human-like referring expressions are not necessarily more helpful ones. Large individual differences are often found in RE production, and it is reasonable to expect that some speakers will be better at giving good instructions than others. Belz and Gatt (2008) compare task-based evaluations (search time and accuracy) to intrinsic ones (string similarity to human models) on computationally generated referring expressions from the ASGRE challenge (Belz and Gatt, 2007) and find no correlation between the two. While this experiment involved simple domains (furniture and people, identified by discrete-valued attributes), it stands as a warning that not all human behavior in REG should be interpreted as facilitating visual search. Thus, the question of ordering preferences for relative descriptions is really two questions: how speakers actually behave, and how they should normatively behave to facilitate visual search for listeners.

REG models which use relative descriptions are often separated into those focused on *identifying* a target object among distractors and those *locating* it in space (Barclay, 2010). We view both of these as strategies for accomplishing the higher-level goal of placing an unknown but visible entity into *common ground* (Clark and Wilkes-Gibbs, 1986), the set of entities which each participant knows is familiar to the other. However, the properties of the domain and task constraints may affect which of these strategies is most appropriate, and therefore what sort of behavior experimenters observe.

In relatively small domains where targets are easy to spot, the primary focus is on identification. When human speakers generate relative descriptions for easy-to-see targets, they mention the landmark after the target, as in the GRE3D7 corpus (Viethen and Dale, 2011), which was specifically set up to elicit relative descriptions using small 3-dimensional images of geometric objects. Models of REG in these kinds of domains (surveyed in Krahmer and van Deemter, 2012) do not emphasize ordering strategies or the need to make syntactic decisions during the planning phase.

Models for visually complex domains such as direction-giving (Barclay, 2010; Gkatzia et al., 2015) must both disambiguate and locate the target. Even when the target is unambiguous, it may still be necessary to use disambiguating descriptions for landmarks (Barclay, 2010). Studies in this kind of domain have followed Talmy (1983) in finding that large, relatively stationary “background” objects make good landmarks for locating an entity rather than simply disambiguating it. For the most part, however, these studies have also focused on what is said (the choice of landmarks and prepositions) rather than the order of mention and the syntactic strategies used to achieve it.

This study extends an earlier one, Elsner et al. (2014), which does look for ordering preferences in human-authored relative descriptions. That study found that larger objects were more likely to be ordered earlier in the description. However, there was no effect on order of mention from a low-level visual salience model, raising potential doubts about whether ordering preferences are truly driven by visual salience. The lack of effect for salience could potentially be due to poor performane of the computational visual salience models: many different salience models have been developed over the last 15 years and there is no agreed on standard, or even a strict defintion of what is meant by low-level salience! Furthermore, our stimuli consisted of cluttered cartoon images which may be problomatic for models trained on photographs of natural scenes. In this study, we re-analyze the same data with a more sophisticated salience model and obtain an improved fit to the data, suggesting that the hypothesized effect of low-level salience is real. Duan et al. (2013), studying the same corpus, find visual effects on determiner selection, and similarly conclude that perception has an impact on late stages of the generation pipeline. These studies focus on generation, leaving open the question of whether the effects they observed were useful to listeners or not.

The question of which speaker behaviors help listeners is tightly connected to the question of whether speakers actively reason about their audience to *try* to help them, a process called *audience design*. Experimental evidence for audience design is widespread. Speakers overspecify descriptions more when they believe the task is important (for example, instructing a surgeon on which tool to use; Arts et al., 2011). They can keep track of which objects they've discussed with a particular listener (Horton and Gerrig, 2002). And they are more likely to tell listeners about an atypical element of an illustrated action (“stabbed with an icepick” vs. “a knife”) when they know listeners can't see the illustration (Lockridge and Brennan, 2002). Audience design is widely accepted as a theoretical assumption underlying neo-Gricean models of reference (Frank and Goodman, 2012; Vogel et al., 2013) and experiments with language games (Degen and Franke, 2012; Rohde et al., 2012). But despite speakers' capabilities for design, not all speaker behavior is audience-driven. Speakers also try to minimize their own effort by mentioning objects and attributes in the order they see them (Pechmann, 1989), avoiding cognitively expensive scanning of irrelevant parts of the scene (Beun and Cremers, 1998), and using their own private knowledge as a proxy for common ground (Horton and Keysar, 1996). Strategies like these make the speaker's task easier, but these savings potentially come at the listener's expense.

Both models offer potential explanations for order-of-mention effects. Pechmann (1989) describes speakers' use of non-canonical adjective orders (“red big”) for visual scenes and argues that such orderings result from an incremental sentence planning strategy (speakers initially perceive the target object's color and only later establish its size relative to other objects in the scene).

Accounts of ordering preferences in non-visual settings usually attribute them to audience design in the form of information-structural principles. Prince (1981) distinguishes between entities which are new to the discourse and those which have previously been mentioned. The first element in an English sentence is generally reserved for old information (already in common ground), while new information is placed at the end (Ward and Birner, 2001, *inter alia*). A variety of non-canonical syntactic constructions, such as *there*-insertion, are analyzed as strategies for enforcing these structural principles. In particular, Maienborn (2001) states that sentence-initial locatives can be *frame-setting* modifiers, which are a type of sentence topic explaining in what context the remaining information is to be interpreted. Information-structural ordering principles can be said to be driven by audience design, since understanding what information is in common ground requires reasoning about the listener. In particular, objects which are clearly perceptually accessible to the listener are treated as familiar (Roberts, 2003).

Thus, the ordering preferences examined here could arise from either mechanism. In an effort-minimization model, speakers talk earlier about large objects because they notice them first. In an audience-design model, speakers talk earlier about large objects because they believe their listeners will notice them first. Thus, either model predicts that more visually salient objects are placed early in the sentence. Our first contribution is to verify that this prediction is in fact true.

The two models differ in their predictions about listener behavior. If the ordering effect is due to effort minimization, it may or may not be helpful for listeners. If it is due to audience design, then (assuming speakers who try to be helpful actually are so), it should facilitate listeners' visual search for the target. Thus, if this ordering principle does not facilitate visual search, it cannot be an audience design effect. Our second contribution is to show that it does in fact facilitate visual search.

## 3. Corpus study

In this section, we test whether speakers prefer to place visually salient landmarks earlier in their referring expressions. The study expands upon Elsner et al. (2014), which used the same corpus of referring expressions, by adding better models of low-level visual salience in order to demonstrate that the effect is actually salience-driven, and includes an additional feature that encodes whether the landmark is spatially located to the left or right of the target in the scene. The procedures for using mixed-effects linear models have also been altered slightly in line with recommendations by Barr et al. (2013).

A relative description of an object has two elements: the *anchor* (the object to be located) and the *landmark* (mentioned only as an aid). Typically the anchor is the *target* of the expression overall, but some REs nest relative descriptions— “the woman next to the man next to the building”— in which case “man” is the landmark relative to “woman” but the anchor relative to “building.”

In a complex image like the scenes from the search-and-find Where’s Wally books (Handford, 1987, 1988, 1993), which were used to elicit descriptions in the corpus study, there can be many ways to describe a particular entity. We distinguish four strategies for ordering the landmark relative to the anchor, which we illustrate with examples from our corpus. The examples below all refer to targets in a Where’s Wally scene showing the construction of a pyramid, with text describing the landmark in italics and text describing the anchor (in these cases also the target) in bold:

- Precede: Directly in front of *the crypt that is green* there is **a man with no shirt and a white wrap on**.
- Precede-establish: Find *the sphinx (half man half lion)*. To the left of *it* is **a guy holding a red vase with a stripe on it**.
- Interleaved: Near the bottom right, **a man walking** beside *the rock* **with his right foot forward**.
- Follow: **The man in a white loincloth** at the upper left of the picture **standing** next to *a bald man*.

These ordering strategies^1^ are distinguished based on the surface order of first mentions in the text. In the precede strategy, the first mention of the landmark occurs before any mention of the anchor. In the precede-establish strategy, the landmark is first mentioned in its own clause, without a relation to the anchor (typically using “there is,” “look,” or “find”), and related to the anchor later. In the interleaved strategy, the anchor is described first, then the landmark, and then the anchor again. In the follow strategy, the anchor is mentioned first, then the landmark.

### 3.1. Dataset and annotation

We analyze a collection of referring expressions for target people in images taken from the Where's Wally childrens picture books (Handford, 1987, 1988, 1993). The dataset^2^ was originally collected by Clarke et al. (2013) in a study showing the effects of perceptual features (clutter and salience) on the selection of landmarks in REs. Mechanical Turk was used to collect the data using a task in which participants were asked to produce descriptions for targets over 11 images. In each image, 16 cartoon people were designated as targets and each participant saw each scene only once, with one of the targets designated with a colored box. The participant was instructed to type a description of the person in the box so that another person viewing the same scene (but without the box) would be able to find them.

**Figure 1:**
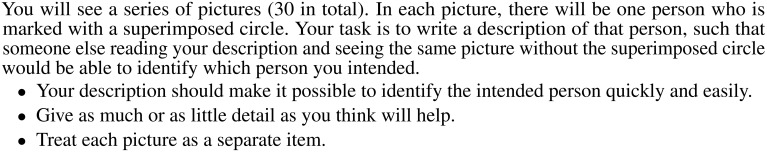
**Instructions for the picture description task in Clarke et al. (2013)**.

The text of the instructions is shown in Figure 1. It asks participants to both identify and locate the target object (and as such is conceptually similar to the “please, pick up the X” frame used in Viethen and Dale, 2011).

Participants were trained on what makes a good referring expression in this domain by carrying out two visual searches based on different descriptions. The dataset contains 1672 descriptions, contributed by 152 different participants.

The REs are annotated for visual and linguistic content. The annotation scheme indicates which substrings of the RE describe the target object, another mentioned object or an image region such as “the left of the picture.” References to parts or attributes of objects are not treated as separate objects; for example, “a man holding a red vase” is a single object. The mentioned objects are linked to bounding boxes (or for very large objects, bounding polygons) in the image. For each mention of a non-target object, the annotation indicates whether it is part of a relational description of a specific anchor, and if so which; if it is not, it receives an establish tag. These annotations are used to determine the ordering strategies used in this study. In some cases, the linkage between objects is implicit:

- … *a group of 11 slaves is following* a slavemaster from left to right across the image. Choose **the third slave in line (the second bald slave)** [ = of the 11 slaves].

In the RE above, the “group of 11 slaves” is introduced with an establish construction, since in that clause, the group is not used as a landmark to locate another object. The group is later used as a landmark (implicitly, via the expression “third slave”). Since the first mention of the group precedes the anchor “third slave,” this is marked precede, and therefore falls into the precede-establish pattern.

### 3.2. Distribution of ordering strategies

Our analysis covers each pair of anchor and landmark mentioned in the corpus (often more than one per description). In all, there are 3290 such pairs in the dataset. As shown in the first row of Table 1, the precede strategies, in aggregate, slightly outnumber the follow strategy; this is due to the overwhelming preference for image regions (“the left”) to precede their anchors. The interleaved ordering is less common, but still quite well-represented.

**Table 1:** **One-vs.-all regression effects predicting order of anchor and landmark in relative descriptions**.

	**PRECEDE**	**PRECEDE-EST**	**INTER**	**FOLLOW**
**% (*n*) Instances**	**28% (918)**	**15% (493)**	**24% (797)**	**33% (1081)**
intercept	2.64	−3.38	−2.44	−5.26
anch area	−0.42^**^	−0.21	−0.22^**^	0.40^**^
anch centr	0.16^*^	X	X	−0.13
anch deps	−0.19	−0.77^**^	0.26^**^	0.11
anch = targ	0.16	−0.32	0.84^**^	−0.80^**^
anch sal	−0.09	0.00	0.00	0.05
distance	0.02	X	X	0.03
sign. lr. dist.	−0.01	X	X	0.01
lmk = reg	15.68^**^	−∞	−∞	−16.42^**^
lmk area	3.97^**^	−0.67	1.53^**^	−4.48^**^
lmk centr	−1.12^**^	−1.03	−0.03	1.37^**^
lmk deps	0.07	1.31^**^	−0.57^**^	−0.75^**^
lmk sal	0.22^**^	0.13	−0.07	−0.17^*^

To verify that this distribution does not simply reflect different participants' differing interpretations of the task description (so that some participants focused only on *identifying* targets while others focused only on *locating* them), we analyze the distribution of strategies within subject. We examine the strategies chosen for all pairs consisting of a target and non-image-region landmark. All but 3 of 152 participants use more than one strategy, and the median number of strategies used is three (of the four total). This shows that subjects selected strategies in a scene- and target-dependent way, and thus variation does not reflect differences across participants in their interpretation of the task.

We conduct four one-vs.-all regression analyses to analyze which factors predict the choice of each order. The factors selected for analysis include measurements of visual salience (the area of the anchor and landmark bounding boxes, their distance to screen center (centr.) (calculated to the center of the object's bounding box), and a low-level salience score indicating pixel dissimilarity from the background. These properties are known to make objects more visually salient and easier to find (Wolfe, 2012), and to increase their chances of being chosen as landmarks (Kelleher et al., 2005; Golland et al., 2010; Clarke et al., 2013). We also include visual factors for the distance between the two objects, and for the signed left-right distance (in case the string ordering is affected by which object appears further left in the image). We also include the number of dependents (landmarks mentioned relative to the object in the description) as a linguistic factor. Large numbers of dependents tend to lead to a “heavier” phrase which is more likely to need its own clause, or to shift to the end of a sentence (White and Rajkumar, 2012). Finally, we include some task-based factors: whether the anchor is the overall target of the expression and whether the landmark is an object or an image region.

The low-level salience score used in this study is a computational measurement of how visually distinctive the object is, based on a comparison of its visual features with the rest of the image. The score used here differs from the Torralba et al. (2006) score used in Elsner et al. (2014), which was not found to be a significant predictor of ordering strategy. In this study, we compute an improved score by reanalyzing the Wally images with five low-level salience models, creating five salience maps for each image. The salience models used were: Achanta (Achanta et al., 2009), AIM (Bruce and Tsotsos, 2007), AWS (Garcia-Diaz et al., 2012), CovSal (Erdem and Erdem, 2013), RCS (Vikram et al., 2012), and SIG (Hou et al., 2012). Images were preprocessed by downsampling by a factor of four. For each salience map, we compute the mean salience within every labeled bounding box in the image. Since the output of the salience models is highly correlated, we then perform PCA (Principal Components Analysis) on the scaled matrix of salience measurements and take the first principal component of the transformed data as a cross-model consensus salience score.

We transform area to square root area and log-transform distance (between objects) and centrality (distance from object to center of image) values. Centrality values are negated, so that higher numbers indicate more central objects. We then scale all continuous factors to zero mean and unit variance and deviation-code binary factors as –0.5, 0.5. We fit a binomial generalized linear model of the data, using uncorrelated random slopes and intercepts for speaker and item (Barr et al., 2013) using LME4 Bates et al. (2015)^3^. No interaction terms were included. Models for precede and follow converged using the default optimization settings. Models for precede-est and inter failed to converge with these settings. For these analyses, image regions were discarded from the dataset (since regions essentially always precede and never use these strategies); the coefficient for this effect is indicated as −∞. Then the effects with the smallest coefficients were removed until convergence; these coefficients are shown as X. Significance of factor main effects was tested using ANOVA to compare a model including all factors and a model leaving out the factor of interest^4^.

Results of the regression analysis appear in Table 1. The largest effects are those relating to image regions, which overwhelmingly occur in the precede order (15.68 precede vs. –16.42 follow). Area of the landmark also has a substantial effect; larger objects tend to precede (3.97) and interleave (1.53) while smaller ones follow (−4.48). Objects with many dependents (“heavy” phrases) occur more often in precede-establish constructions (1.31) and less often in interleave and follow (–0.057, –0.075).

Smaller, but still significant, effects include anchor area; larger anchors are less likely to be preceded by landmarks (–0.42) and more likely to be followed (0.40). The target is more likely to interleave around a landmark (0.84). Finally, the low-level salience score has slight effects for landmarks, but not for anchors: more visually distinctive landmarks are more likely to precede their anchors (0.22) and less likely to follow them.

No significant effect is found for either distance measurement.

### 3.3. Analysis

The strong effects of anchor and landmark area support the hypothesis that more visually salient objects are considered part of common ground and that speakers place them earlier in their descriptions. The effects of the low-level salience score, though weak, point in the same direction. The effects of centrality are counterintuitive (more central landmarks are less likely to precede). This pattern is difficult to explain, since increasing centrality normally makes objects more salient (Judd et al., 2012). We speculate that the effect might be due to the frequent use of region descriptors like “at the top right” to restrict attention to off-centered areas of the image.

While the low-level salience score has a significant effect, its contributions are minor. This may indicate that area, rather than overall visual salience, is indeed the major contributing factor for ordering. But this explanation fits poorly with both visual and linguistic theories, since it posits a special-case visual process and an exception to our usual understanding of how objects enter common ground. A better explanation is probably that computational salience modeling simply does not capture all the complex factors which make up visual distinctiveness in a domain like Where's Wally. Clarke and Keller (2014) show that many popular low-level salience models fail to account for viewer perceptions even in simple contrived stimuli. Thus, the composite score used in this analysis is likely capturing only some of the visual distinctiveness of objects in the scene.

The primary motivation for the precede-establish construction appears to be linguistic; it occurs when the landmark itself has many dependent sub-landmarks and thus requires its own clause. It is less likely to be chosen if the anchor is large and easily spotted on its own (in which case the preferred order is follow). But it is also not as often selected for large landmarks (which don't require dependent sub-landmarks or their own clause). These findings are in accord with Ward and Birner (1995), who state that objects introduced by existential “there is” should be new to the discourse. The establish strategy is a way of putting these important but hard-to-see landmarks on the left of the clause without marking them as common-ground information.

## 4. Perception study

If speakers prefer to use the precede order for easier to find (larger and more salient) landmarks vs. the follow order for harder to find (smaller and less salient) ones, do these tendencies help listeners to find the target objects quickly? We conduct a visual search experiment using the Wally images and controlled linguistic stimuli to evaluate this hypothesis. Since area, centrality and low-level distinctiveness models gave equivocal results as proxies for visual salience in the previous section, in this experiment, we measure visual salience more directly. We use target-only and landmark-only visual search tasks as indicators of how easy each object is to see on its own, and analyze the relative descriptions in the context of these scores for their components.

### 4.1. Stimuli

Stimuli consist of a Where's Wally image paired with a referring expression. There are four conditions, illustrated with examples referring to a Wally scene involving the construction of a pyramid. We selected a single target and landmark in each image, so that the objects and attribute-based descriptions used in the Target and Landmark stimuli for a given scene also feature in the Landmark precedes and Landmark follows stimuli:

- Target: At the upper right, **the man holding the red vase with a stripe**.
- Landmark: At the upper right, *the sphinx*.
- Landmark precedes: At the upper right, to the left of *the sphinx*, **the man holding the red vase with a stripe on it**.
- Landmark follows: At the upper right, **the man holding the red vase with a stripe** to the left of *the sphinx*.

The targets and landmarks are chosen to represent a range of relative size and perceived visual salience values, and to be approximately balanced across regions of the screen. In each case, the target person is one of the people used as targets in Clarke et al. (2013); when possible, the landmark is also one mentioned by speakers in the corpus, although in a few cases this was not possible since speakers did not mention a landmark of the desired size. Descriptions of targets and landmarks contained enough attributes to make them unambiguous in isolation (so that a relative description was an overspecification, not the only disambiguating detail).

All stimuli were read by a British English speaker. Recordings in the *landmark* condition are the fastest (mean length 2.6 s) followed by the *target* condition (3.0 s). The relative description cases are longer and therefore slower; when the landmark precedes, the mean length is 4.4 s while when it follows, the mean length is 4.2.

### 4.2. Experimental procedures

The experiment was conducted in the Eye Movements and Attention laboratory at the University of Aberdeen. Experimental scripts were created and run using MatLab and run on a PowerMac. Stimuli were presented on a 61 cm Sony Trimaster EL computer screen, 1080 × 1920 computer screen. Participant responses were recorded using an Apple keyboard and mouse. An EyeLink 1000 was used to conduct eye-tracking, although eye-movements are not analyzed here. The protocol for each of the experiments was reviewed and approved by the Psychology Ethics Committee at the University of Aberdeen.

Thirty-two participants (median age 23, range = 19–42 years old, 21 females) took part in the study. Participants were recruited from the population of students and other members of the academic community at the University of Aberdeen. All participants had normal or corrected-to-normal vision and were native English speakers. The experiment was conducted with the full understanding and signed consent of each participant. Participants were remunerated $ 5–10 for their time, depending on the number of experiments they had taken part in.

Immediately following image onset, an audio recording of the search instruction was played to participants over headphones, giving them the necessary information required to find the target. Participants pressed the space bar on the keyboard when they had found the specified target. They were then required to use the mouse to click on the target. This was done so that we had a record of search accuracy and participants were not able to just press space without finding the target. Reaction time was recorded as the time from image onset to when the space bar had been pressed. There was no requirment for the participant to listen to the whole referring expression.

### 4.3. Outliers

The complete dataset consists of 896 trials (32 × 28). We filter the reaction time data from the perception study by discarding instances where the listener failed to find the target, or incorrectly signaled success before actually finding it. A single participant was discarded for excessively long reaction times. All trials for which the reaction time recorded was < 0.5 s or >10 s were discarded, as were trials for which the time between the keypress signaling successful detection and the click to indicate the found item was greater than 5 s. These filters exclude 186 trials after which 669 remain. A software error prevented measurement of the click location for 56 trials, so we have accuracy information for only 613 of these.

### 4.4. Results

Overall, participants reacted faster to the non-relative expressions (median 3.9 s for targets and 3.7 for landmarks) than the relative ones (4.6 s for target-first REs and 4.9 for landmark-first REs). These times are approximately a second longer than the stimuli, and indicate that our visual search task was reasonably easy, especially given the cluttered nature of the scenes. In particular, the short search times for target-only expressions demonstrate that the relative descriptions were truly overspecified, since participants could find the targets without them. As usual in complex visual search tasks, standard deviations are substantial (between 1.0 and 1.3 for all cases).

Our analysis focuses on comparisons between the two orders for relative REs (precede and follow). We hypothesize that, when the target is easier to find than the landmark, search is facilitated by landmark following the target, while when the landmark is easier, search is facilitated by the landmark preceding. We separate the stimuli into three categories, “target-easier,” “target-harder,” and “both-similar,” based on the empirical reaction times for the target-only and landmark-only cases. For each image, we compute:


(1) $$\begin{array}{r} Z\left(median\left(rt_{targ-only}\right)-median\left(rt_{lmark-only}\right)\right) \end{array}$$

This is a Z-transformed score of how much easier it is for participants to find the target than the landmark. We select the bottom third (nine instances) as “target-easier,” the middle third (9 instances) as “both-similar,” and the upper third (10 instances) as “target-harder.”

**Figure 2:**
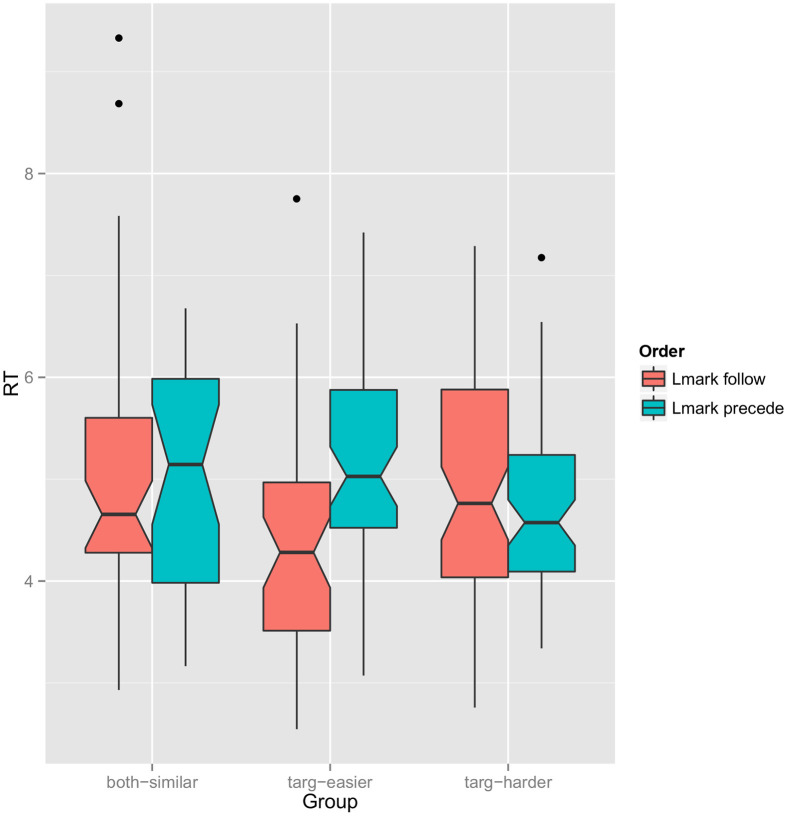
**Notched boxplot of reaction time as a function of referring expression order (red: target first, blue: landmark first) grouped by which object is easier to find**. Notches represent 95% confidence interval of the median (computed with GGPlot default settings).

Figure 2 shows a plot of reaction time as a function of referring expression order within each group. Median RTs are lower for the landmark follow order in the “both-similar” and “target-easier” groups and higher in the “target-harder” group. The overall median RT for the relative referring expressions is 4.7 s. In the “target-easier” group, the median for follow expressions is 4.3 while for precede expressions it is 4.9. For the “target-harder” group, the median for follow expressions is 5.3 while for precede expressions it is 4.7.

We perform the Mann-Whitney test for differing medians on each group. For the “both-similar” group, the test fails to find significance (*p* > 0.05); for the “target-easier” group, *p* < 0.01 and for the “target-harder” group, *p* < 0.05^5^.

**Figure 3:**
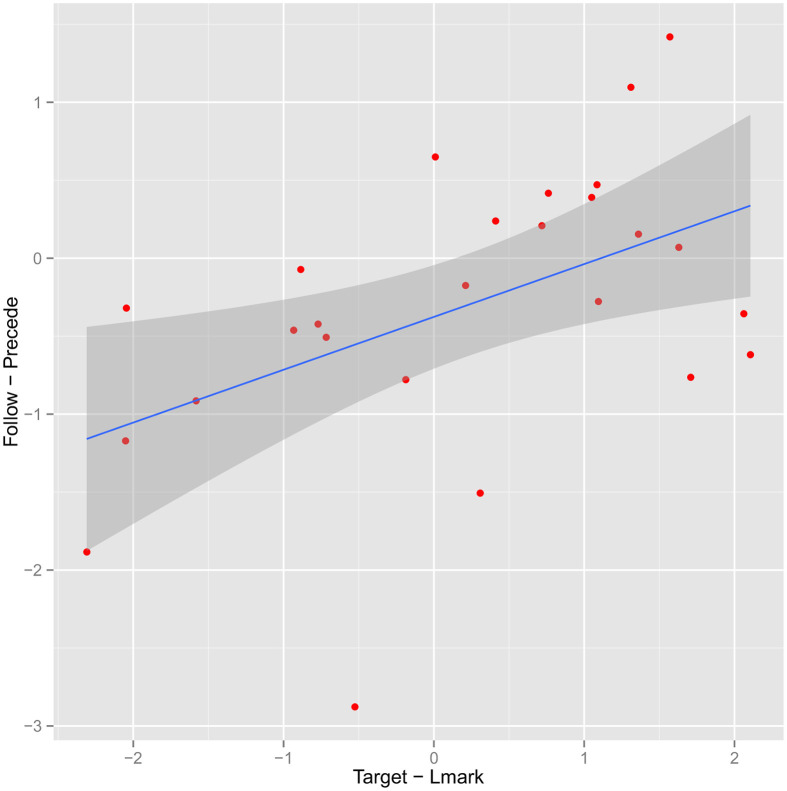
**Plot of median (target-first-landmark-first) reaction time as a function of median (target-landmark) reaction time**. Each point represents a stimulus; fitted regression line uses linear model.

In addition to this analysis based on grouping the items, it is also possible to look at the median (*target* − *lmark*) (Equation 1) as a continuous predictor. In Figure 3, we plot it against the analogous quantity for the two relative referring expressions, median (*follow* − *precede*). Points on the left represent instances where the target is found faster than the landmark in isolation. Points at the bottom represent instances for which the follow order leads to a faster search. Thus, our hypothesis would predict a positive correlation. The estimated Pearson linear correlation is 0.52, (95% confidence interval 0.17–0.75).

Participants are relatively accurate (of 613 cases with accuracy information, 487 found the correct item with an error < 150 pixels on either axis). We checked for an accuracy effect by group similar to the effect on reaction times, but there is none. Unsurprisingly, the majority of identification errors for relative descriptions (62 of 77) occur in the “target-harder” group, indicating that when the target takes longer to find, it is also more likely to be misidentified. But these are distributed evenly across the two RE orders^6^.

### 4.5. Discussion

Under both analyses of the visual search study, the results are as predicted by our hypothesis: search is facilitated by mentioning the easier-to-find object first. The difference in medians suggests an average effect of about 0.6 s in either direction. Since the reaction time is measured from the start of the utterance, the results imply that giving the target description later in the trial can sometimes be beneficial, even though listeners in this condition must wait longer before they can possibly react.

Several caveats apply. First, although we find the expected facilitation effect when comparing among differently ordered relative descriptions, overall, participants reacted faster to the *non*-relative (target-only) expression. Even for the “targ-harder” group, mentioning the target alone yields a median search time of 4.2 s, while a relative description with the landmark first yields a median of 4.7.

If target-only descriptions actually lead to faster search than relative ones, why use a relative description at all? Clarke et al. (2013) show that relative descriptions are extremely common in human REs for these scenes, an effect also shown in a variety of previous work (Viethen and Dale, 2008). Overspecification is often intended to ensure the listener that they have actually found the right object (Arts et al., 2011; Koolen et al., 2011). If the listener believes confirmatory information is coming, they may wait to be sure they find the right object. However, Listeners are no more accurate in these conditions.

Secondly, the analysis does not correct for possible per-participant or per-item effects. This is partly due to the small amount of data, and partly to the use of median statistics to group the items as easier or harder. Since no participant heard more than one condition for a given stimulus, the easier/harder grouping reflects data from different participants than the reaction times plotted for relative descriptions within that group, complicating any analysis of individual differences.

## 5. Conclusion

Our analysis finds evidence for both of our hypotheses: speakers treat visually salient landmarks as being in common ground, preferring to place them early in their descriptions, and this ordering principle aids listeners in finding the target of a relative description quickly. These findings remain consistent with an audience-design model of perceptual effects in REG. In other words, speakers keep mental track of which objects in the scene are easier or harder to perceive. They use this information to preferentially select easier-to-see objects as landmarks, and they treat easier- and harder-to-see landmarks differently when planning the syntax of their descriptions. Both of these tendencies stem from the desire to make sure their listeners can efficiently find the object they are trying to point out.

While the results are consistent with such a model, we should emphasize that they do not rule out a least-effort model in which speakers talk more about things they themselves see earlier. To eliminate this possibility, we could give the speaker and listener different views of the scene [for instance, by occluding part of the scene for the listener (Brown-Schmidt et al., 2008)]. Alternately, we could look more closely at the time course of REG, using eye-tracking to determine when speakers discover the objects they mention and how much planning time intervenes.

Our findings definitely indicate that the choice of ordering strategy must be sensitive to visual features and cannot simply be left to an off-the-shelf micro-planning and realization component. This differentiates it from purely surface phenomena like dependency length minimization and heavy NP shift, which can be implemented at a late stage of the pipeline White and Rajkumar (2012). Choosing the correct strategy has a modest, but significant impact on listener performance. We find differences of about 0.6 s for referring expressions of about 4.7 s in length; in other words, the median subject's search will be about 10% easier if the correct ordering is used. Since we also found that relative descriptions lead to slower searches in general, this result should be considered with some caution. The stimuli used in this study were deliberately overspecified so that subjects could find the appropriate object using the non-relative description alone. Real relative descriptions are not always overspecified, but might be necessary to disambiguate the target; in these cases, they will presumably not cause a slowdown. The direction and magnitude of the slowdown effect might also vary depending on the complexity and visual clutter of the scene. Nonetheless, we believe that new REG systems should use perceptual information to properly order the relative descriptions they generate.

Our findings show that seemingly low-level and disparate mental “modules” like perception and sentence planning interact at a high level and in task-dependent ways. But we have yet to determine what sort of mental representations these systems use to communicate, or what underlies the considerable variation we find among both speakers and listeners. Our datasets are too small to tell us whether this variation reflects different populations, each using different strategies, or whether there is comparable variation within a single individual. Nor can it tell us whether larger-scale cognitive differences (for example, in attention, memory, or executive function) could account for these differences.

### 5.1. Data sharing

The referring expressions used in the corpus study are publically available as the WREC (Wally Referring Expression Corpus): http://datashare.is.ed.ac.uk/handle/10283/337. See Clarke et al. (2013). The recorded stimuli used in the comprehension experiment are provided as Supplementary Material to this paper.

## Author contributions

AC and ME designed the visual search comprehension study, which was run by student research assistants under the supervisor of AC. HR and ME selected and recorded the language stimuli. Analysis of the results was carried out by ME and AC. ME implemented the statistical analyses, while AC provided the visual salience information. The manuscript was jointly written by AC, ME, and HR.

### Conflict of interest statement

The authors declare that the research was conducted in the absence of any commercial or financial relationships that could be construed as a potential conflict of interest.

## Footnotes

^1^There are also six examples of establish constructions without the precede order, which we discard from further analysis.

^2^Released as the Wally Referring Expressions Corpus (WREC): http://datashare.is.ed.ac.uk/handle/10283/337.

^3^In LME4, the model is specified as *follow* ~ *area* + (0 + *area*|*speaker*) + (0 + *area*|*image*) + … + (1|*speaker*) + (1|*image*).

^4^*P*-values are presented without the Bonferroni correction for multiple comparisons. A set of 52 comparisons at the 0.05 level includes about three type II errors on average.

^5^The null hypothesis for the “target-harder” medians cannot be rejected at a Bonferroni-corrected level of 0.05∕3 = 0.016.

^6^We also ran the analyses above excluding trials on which a misidentification occurred; results are qualitatively similar, except that the test of whether median RTs differ in the “target-harder” group cannot be rejected.
