# Corrigendum: Giving good directions: order of mention reflects visual salience

**DOI:** 10.3389/fpsyg.2024.1444968

**Published:** 2024-07-19

**Authors:** Alasdair D. F. Clarke, Micha Elsner, Hannah Rohde

**Affiliations:** ^1^School of Psychology, The College of Life Sciences and Medicine, University of Aberdeen, Aberdeen, UK; ^2^Department of Linguistics, The Ohio State University, Columbus, OH, USA; ^3^Linguistics and English Language, University of Edinburgh, Edinburgh, UK

**Keywords:** referring expressions, visual search, visual salience

In the published article, there was an error in Figure 1 as published. The license to use the Where's Wally image has expired. In the updated article, this figure has been removed. The remaining figures have been renumbered accordingly, and the text has been amended as outlined below to remove the previous figure citations.

A correction has been made to **3. Corpus Study**, Paragraph 3. Instead of “In a complex image like the scenes in Where's Wally (see Figure 1), there are many ways to describe a particular entity. We distinguish four strategies for ordering the landmark relative to the anchor, which we illustrate with examples from our corpus (all referring to targets in Figure 1), with text describing the landmark in italics and text describing the anchor (in these cases also the target) in bold:,” the corrected text is shown below.

“In a complex image like the scenes from the search-and-find Where's Wally books (Handford, 1987, 1988, 1993), which were used to elicit descriptions in the corpus study, there can be many ways to describe a particular entity. We distinguish four strategies for ordering the landmark relative to the anchor, which we illustrate with examples from our corpus. The examples below all refer to targets in a Where's Wally scene showing the construction of a pyramid, with text describing the landmark in italics and text describing the anchor (in these cases also the target) in bold:”

A correction has also been made to “*3.1. Dataset and Annotation*,” Paragraph 4. Instead of “References to parts or attributes of objects are not treated as separate objects; “a man holding a red vase” in Figure 1 is a single object.” The corrected sentence should be “References to parts or attributes of objects are not treated as separate objects; for example, “a man holding a red vase” is a single object.”

A correction has been made to “*3.1. Dataset and Annotation*,” Paragraph 1. Instead of “In each image, 16 cartoon people were designated as targets and each participant saw each scene only once, with one of the targets designated with a colored box, as shown in Figure 1.” The corrected sentence should be “In each image, 16 cartoon people were designated as targets and each participant saw each scene only once, with one of the targets designated with a colored box.”

A correction has been made to “*4.1. Stimuli*,” Paragraph 1. Instead of “There are four conditions, illustrated with examples referring to Figure 1.” The corrected sentence should be “There are four conditions, illustrated with examples referring to a Wally scene involving the construction of a pyramid.”

The authors apologize for these errors and state that they do not change the scientific conclusions of the article in any way. The original article has been updated.
